# Beneficial effects of curcumin nano-emulsion on spermatogenesis and reproductive performance in male rats under protein deficient diet model: enhancement of sperm motility, conservancy of testicular tissue integrity, cell energy and seminal plasma amino acids content

**DOI:** 10.1186/s12929-017-0373-5

**Published:** 2017-09-02

**Authors:** Omar A.H. Ahmed-Farid, Maha Nasr, Rania F. Ahmed, Rofanda M. Bakeer

**Affiliations:** 1 Department of Physiology, National Organization for Drug Control and Research, Giza, Egypt; 2 Deparment of Pharmaceutics, Ain Shams University, Cairo, Egypt; 3 Department of Pharmacology, Medical division, National Research Centre (ID: 60014618), Giza, Dokki 12622 Egypt; 4 Department of Pathology, Medical division, National Research Centre (ID: 60014618), Giza, Dokki 12622 Egypt

**Keywords:** Protein deficient diet, Curcumin, Male fertility, Nano-curcumin

## Abstract

**Background:**

Malnutrition resulting from protein and calorie deficiency continues to be a major concern worldwide especially in developing countries. Specific deficiencies in the protein intake can adversely influence reproductive performance. The present study aimed to evaluate the effects of curcumin and curcumin nano-emulsion on protein deficient diet (PDD)-induced testicular atrophy, troubled spermatogenesis and decreased reproductive performance in male rats.

**Methods:**

Juvenile rats were fed the protein deficient diet (PDD) for 75 days. Starting from day 60 the rats were divided into 4 groups and given the corresponding treatments for the last 15 days orally and daily as follows: 1st group; curcumin group (C) received 50 mg/kg curcumin p.o. 2^nd^group; curcumin nano-form low dose group (NCL) received 2.5 mg/kg nano-curcumin. 3rd group; curcumin nano-form high dose group (NCH) received 5 mg/kg nano-curcumin. 4th group served as malnutrition group (PDD group) receiving the protein deficient diet daily for 75 days and received distilled water ingestions (5 ml/kg p.o) daily for the last 15 days of the experiment. A normal control group was kept under the same conditions for the whole experiment and received normal diet according to nutrition requirement center daily for 75 days and received distilled water ingestions (5 ml/kg p.o) daily for the last 15 days of the experiment.

**Results:**

PDD induced significant (*P* < 0.05) reduction in serum testosterone level, sperm motility, testicular GSH, CAT, SOD, testicular cell energy (ATP, ADP and AMP), essential and non-essential amino acids in seminal plasma, an increase in testicular MDA, NOx, GSSG and 8-OHDG. Data was confirmed by histological examination and revealed pathological alteration in the PDD group. Ingestion of curcumin (50 mg/kg) and curcumin nano-emulsion (2.5 and 5 mg/kg) showed significant (*P*< 0.05) amelioration effects against PDD-induced disrupted reproductive performance as well as biochemical and pathological alterations and the overall results of the nano-emulsion (5 mg/kg) were comparable to curcumin (50 mg/kg).

**Conclusions:**

The present study suggests that administration of curcumin nano-emulsion as a daily supplement would be beneficial in malnutrition- induced troubled male reproductive performance and spermatogenesis cases.

## Background

Nutrition plays an important role in growth and development of the reproductive system. Evidence that reproductive maturation and function are influenced by malnutrition is now emerging from animal studies and human populations. It is clinically known that among the several forms of under-nutrition, mild-to-moderate protein and/or energy malnutrition is the most common and frequently impairs the growth and development of reproductive system. A huge number of reports have been published relating protein-calorie malnutrition (PCM) in males to several hazardous health complications and it is well established that this problem probably dominates in over populated countries [1–3].

Curcumin (1,7-bis (4-hidroxy-3-methoxyphenyl)-1,6-hepadiene-3,5-dione), obtained from *Curcuma longa* L. (Zengiberaceae family) rhizomes, has been extensively used in ethnic medicine for centuries and has displayed a wide range of physiological and pharmacological activities [4]. It is present in the commonly consumed foodstuff and considered safe and is known to have antioxidant, anti-inflammatory and immunodulatory properties. Numerous researches suggest the protective action of curcumin against oxidative stress-mediated cardiomyopathy, neuropathy, nephropathy, hepatic injury and testicular dysfunction [5–7].

From the pharmaceutical point of view, there are many challenges that limit the clinical application of curcumin [8, 9], among which is its poor aqueous solubility, photo-degradation, chemical instability, rapid metabolism and short half-life, leading to poor bioavailability when it is administered as such [10]. Therefore nano-systems for curcumin provide a very efficient solution for the aforementioned problems, leading to enhancement of its in vivo bioavailability and maximization of its therapeutic potential. Nano-emulsions which are nano-carriers composed of oil, surfactant/co-surfactant and an aqueous phase represent a very promising delivery system for curcumin as indicated by previous studies, which reported about 9 folds increase in oral bioavailability and better pharmacokinetic profile for curcumin upon encapsulation in nano-emulsion form [11, 12].

Effect of ingesting such nano-emulsions on male fertility has not been experimentally explored before and hence the present study aimed to evaluate the efficacy of curcumin as contrasted to curcumin nano-emulsion (nano-curcumin) in ameliorating protein deficient diet model-induced testicular atrophy, troubled spermatogenesis and decrease reproductive performance in male rats.

## Methods

### Animals

Male Wistar albino juvenile rats weighing 60–70 g were obtained from the animal house colony of Faculty of Pharmacy, Ain Shams University (Cairo, Egypt) and were housed in the National Research Centre (Dokki, Giza, Egypt) animal house in standard polypropylene cages and kept under adequate environmental conditions with equal light − dark cycles.

### Ethics statement

The protocol for the conducted animal experiments was approved by the Research Ethics Committee of the Faculty of Pharmacy, Ain Shams University which followed the recommendations in the Guide for the Care and Use of Laboratory Animals of the National Institutes of Health (NIH publication no. 85–23, revised 1996).

### Chemicals and drugs

Curcumin was purchased from Sigma Aldrich, Germany. Tween 80 was purchased from El-Nasr Pharmaceutical Company (Cairo, Egypt). Labrafac PG oil was a kind gift from Gattefosse’ Company, France.

### Preparation of curcumin nanoparticles

Curcumin was added to amber glass vials containing appropriate amounts of Labrafac PG oil and Tween 80 at a ratio of 1:1, then placed in a thermostatically controlled shaking water bath at 37 °C for 72 h at 100 rpm (Kottermann GmbH, Uetze/Hanigsen, Germany) to reach equilibrium. Preparation of the nano-emulsion was carried out using the spontaneous emulsification method [13], which was carried out by the portion-wise addition of the vial contents to double distilled water with continuous magnetic stirring for 2 h (Yellow line stirrer, IKA, Germany), for the formation of the o/w nano-emulsion. The nano-emulsion was characterized by measuring its particle size using the Zetasizer device (Nano ZS, Malvern instruments, Worcestershire, UK) after appropriate dilution. Its polydispersity index and surface charge was also measured using the same device [14].

### Experimental design

Rats were divided into five groups (*n* = 8), they were fed protein deficient diet (PDD) consisting of pellets of shelled corn grains for 75 days [15, 16]. Drug treatments started from day 60 and for 15 days; rats were divided as follows: **(1). PDD control:** given daily 5 ml/kg distilled water ingestions. **(2). Curcumin group (C):** administered curcumin (50 mg/kg, orally) [17]. **(3, 4). Nano-curcumin groups (NCL, NCH):** respectively administered nano-curcumin (2.5 and 5 mg/kg body weight, orally). A group of 8 rats was kept in a separate cage in the same room, under the same conditions and fed normal standard recommended rats’ pellet diet for 75 days to serve as **Normal control.** Starting from day 60 this group received daily 5 ml/kg distilled water ingestions for 15 days. On day 76; 24 hs. after the last drugs ingestions; blood samples were collected and then rats were sacrificed by decapitation, testes and epididymides were collected. Tissues were either used for biochemical analyses or fixed in 10% formalin for histochemical studies.

### Sample preparation

The testes and epididymides were gently excised. Each testis was weighted and homogenized in saline (10% *w*/*v*); samples were centrifuged at (4500 rpm) for 15 min, the supernatant was isolated and stored at −20 °C until assay. Each epididymal caudal was minced by using sharp scissors to release sperm in 1 ml of PBS (pH = 7.4). The liquefied semen samples were centrifuged at 600 xg for 20 min at 4 °C. The supernatant seminal plasma were separated and stored at −20 °C until assay.

### Mass motility study

A drop of freshly collected semen was placed on a slide kept near body temperature (37–38 °C) and was examined under low magnification (X120), motility of semen samples were rated according to the vigor of the motility of sperms as follows: **(0)** Seminal samples showing no movement. **(1)** Seminal samples showing very slow movement. **(2)** Seminal samples showing slow movement. **(3)** Seminal samples showing moderate movement. **(4)** Seminal samples showing vigorous movement.

### Percentage of progressive motility of spermatozoa

Immediately after each collection, it was assessed by microscopic examination by placing a small drop of fresh semen on a clean warm glass slide (37 °C), diluted with two drops of warm 0.9% NaCl and covered with a cover slip. Examination is made under the high power (X400).

### Percentage of normal spermatozoa

To determine sperm vitality, 40 μl of freshly liquefied semen was thoroughly mixed with 10 μl of eosin Y (1℅), and 1 drop of this mixture was transferred to a clean slide. At least 200 sperms were counted. Sperms that were stained pink or red were considered dead, and the unstained sperms were considered viable. The percentage of normal sperm was calculated.

#### Sperm cell concentration

500 μl of the sperm suspension was diluted with formaldehyde fixative (10% formalin in PBS). Approximately 10 μl from the diluted solution was transferred into a haemocytometer and let to stand for 7 min. Then the settled sperms were counted and evaluated per 250 small squares of a haemocytometer [18–21].

## Biochemical analysis

### Determination of seminal plasma essential and non-essential amino acids levels

Amino acids of seminal plasma were determined using HPLC according to the method of Saunders et al. (1988) with some modifications where; the samples were clarified by centrifugation, 400 μl of seminal plasma supernatant were evaporated under reduced pressure and resuspended in 100 μl of coupling buffer composed of acetonitrile: ethanol: tri-ethylamine: water (10: 5: 2: 3) and then re-evaporated under reduced pressure. Derivatization by Phenyl isothiocyanate (PITC) was accomplished by resuspending the samples in 90 μl coupling buffer plus 10 μl of PITC and incubated for 5 min at room temperature, after that the samples were evaporated, and then resuspended in 240 μl of 50 mM ammonium acetate buffer + 10 μl methanol, pH (6.5). A 20 -μ1, sample was used for HPLC analysis with an Agilent HP 1200 series HPLC apparatus (USA) as described above. Separation of amino acids was conducted using an ultrasphere C18 reversed phase column at wave length 254 nm with UV detector. The mobile phase was a seven step gradient of increasing concentrations of solvent B from 5 to 70%. Solvent A was composed of 50 mM ammonium acetate (pH 6.5) and solvent B was 100 mM ammonium acetate (pH 6.5): acetonitrile (1:1). Flaw rate was 2 ml/min at temperature 50^0^ C [22].

### Determination of serum testosterone level (ng/ml)

Serum testosterone level was determined by ELISA (Enzyme Linked Immunosorbant Assay) kit; obtained from Fortrees Diagnostic Limited, United Kingdom and north Ireland.

### Determination of the testicular tissue GSH and GSSG levels (μmol/g tissue) by HPLC

The thiols compounds of oxidized and reduced glutathione were detected by HPLC system of Agilent HP 1200 series (USA) that consisted of quaternary pump, a column oven, Rheodine injector and 20 μl loop, UV variable wavelength detector. The report and chromatogram taken from Chemstation program purchased from Agilent. 30 cm × 3.9 mm C18 μBondapak column was used. The flow rate was 1 ml/min and UV detection at wavelength 190 nm was applied. 0.0025 M sodium phosphate buffer, pH 3.5, containing 0.005 M tetrabutylammonium phosphate and 13% methanol was used as mobile phase. Samples were compared to glutathione (oxidized and reduced) reference standard purchased from Sigma Chemical Co. The results were expressed as μmol/g tissue [23, 24].

### Determination of the testicular tissue MDA level (nmol/g tissue) by HPLC

For determination of malondialdehyde (MDA) levels; the samples were analyzed on an Agilent HP 1200 series HPLC apparatus (USA) as described above. The analytical column was Supelcosil C18 (5 μm particle and 80 A^o^ pore size) (250 × 4.6 ID). The mobile phase was 82.5:17.5 (*v*/v) 30 mM monobasic potassium phosphate (pH 3.6)–methanol and the flow rate was 1.2 ml/min, wavelength 250 nm was applied for detection. MDA standard was prepared by dissolving 25 μl 1,1,3,3 tetraethoxypropane (TEP) in 100 ml of water to give a 1 mM stock solution. Working standard was prepared by hydrolysis of 1 ml TEP stock solution in 50 ml 1% sulfuric acid and incubation for 2 h at room temperature. The resulting MDA standard of 20 nmol/ml was further diluted with 1% sulfuric acid to yield the final concentration of 1.25 nmol/ml to get the standard for the estimation of total MDA [25–27].

### Determination of the testicular tissue NOx level (μmol/g tissue) by HPLC

Nitrates + nitrites (NO_x_) level was determined using Agilent HP 1200 series HPLC apparatus (USA) as described above. The analytical column was anion exchange PRP-X100 Hamilton, 150 × 4.1 mm, 10 μm. The mobile phase was a mixture of 0.1 M NaCl - methanol, at a volume ratio 45:55.The flow rate of 2 ml/min, wavelength adjusted to 230 nm. The resulting chromatogram identified the concentration from the sample as compared to that of the standard purchased from Sigma Aldrich [28].

### Determination of the testicular tissue CAT activity (U/mg protein) by spectrophotometer

Catalase activity was measured by spectrophotometric method based on the decomposition of H_2_O_2_ [29].

### Determination of the testicular tissue SOD activity (U/mg protein) by spectrophotometer

SOD activity was assayed for 2 min interval. Activity was expressed as the amount of enzyme that inhibits the auto oxidation of pyrogallol and was expressed as U/mg protein. [30]

### Determination of the testicular tissue 8-OHDG content (pg/g tissue) by HPLC

The separation of 8-hydroxy-2-deoxyguanosine (8-OHDG) was performed with an Agilent HP 1200 series HPLC apparatus (USA) as described above. The analytical column was Supelcosil C18 (5 μm particle and 80 A^o^ pore size) (250 × 4.6 ID). The eluting solution was H_2_O/methanol at a ratio (85: 15) with 50 mM KH_2_PO_4_, pH 5.5 at a flow rate of 0.68 ml/min. The UV detector was set at 245 nm. The resulting chromatogram identified the concentration from the sample as compared to that of the standard purchased from Sigma Aldrich [31].

### Determination of testicular tissue ATP, ADP and AMP contents (μg/g tissue) by HPLC

The separation of tissue adenosine tri, di and mono phosphate (ATP, ADP and AMP) was performed with an Agilent HP 1200 series HPLC apparatus (USA) as described above. The analytical column was Ultrasphere ODS EC 250 × 4.6 mm column. Mobile phase A consisted of 0.06 mol/l K_2_HPO_4_ and 0.04 mol/l KH_2_PO_4_ dissolved in deionized water and adjusted to pH 7.0 with 0.1 mol/l KOH, while mobile phase B consisted of 100% acetonitrile. Flow rate of the mobile phase was 1.2 ml/min. ATP, ADP and AMP in the samples were identified by comparison with standards purchased from Sigma Aldrich. The report and chromatograms were taken from chemstation program at wave length 254 nm [32, 33].

Total adenylate energy charge (AEC) was calculated according to the equation [34]:

AEC = (ATP + 0.5ADP)/(ATP + ADP + AMP)

### Histopathological examination

For a variety of reasons, the testis presents a problem for good fixation. All of the regulatory guidelines relating to reproductive studies recommend that the testes should be fixed in Bouin’s or a comparable fixative for observing cellular details as meiosis, mitosis or even apoptosis. Since our study is concerned with normal spermatogenesis not fertility leveling; our samples were fixed in formalin, because formalin fixation of rodent testes results in better preservation of structure than with Bouin’s [35]. Specimens were taken from all groups subjected to our study, which were sliced and fixed in 10% buffered formalin. Paraffin blocks were prepared from those samples after a serial of dehydration, clearing and embedding. The paraffin-embedded material was prepared in 5-μm-thick slices, which were mounted on microscope slides and stained with hematoxylin and eosin and examined by optical microscopy to evaluate the morphologic aspects.

N.B. Minimal artifacts were produced due to formalin fixation.

#### Image Morphometry

Morphometric studies on H&E stained slides were performed using the Leica Qwin 500 Image Analyzer (LEICA Imaging Systems Ltd., Cambridge, England,) which consists of Leica DM-LB microscope with JVC color video camera attached to a computer system Leica Q 500IW.

### Morphometric measurements

#### Detection of circumferences (μm)

Means of seminiferous tubules circumferences of the tested groups were determined by lining of transitionally cut seminiferous tubules (*n* = 8) selected on each field at a magnification of 50X (longitudinal cuts were excluded).

#### Total sperm cells maturation count

Counting of spermatogenic cells were performed including spermatogonia, primary & secondary spermatocytes, spermatids and spermatozoa.

#### Modified Johnsen spermatogenesis scoring

(1) No seminiferous epithelium, (2) No germinal cells, Sertoli cells only, (3) Spermatogonia only, (4) No spermatozoa or spermatids, few spermatocytes, (5) No spermatozoa or spermatids, many spermatocytes, (6) No spermatozoa, no late spermatids, few early spermatids, (7) No spermatozoa, no late spermatids, many early spermatids, (8) Less than five spermatozoa per tubule, few late spermatids, (9) Slightly impaired spermatogenesis, many late spermatids, disorganized epithelium, (10) Full spermatogenesis [36].

### Statistical analyses

Statistical analyses for Total sperm cells maturation count and Modified Johnsen spermatogenesis scoring were carried out using Kruskal-Wallis test followed by Dunn’s multiple comparisons test. All other parameters measured were carried out using one way ANOVA followed by Tukey’s multiple comparisons test using Graph prism software (version 6); where *P* < 0.05 was accepted as being significant in statistical tests. Values were expressed as means ± S.E. Pearson’s correlations studies were conducted using SPSS software (version 17) where *P* < 0.05, *P* < 0.01 and *P* < 0.001 were used to designate the degree of correlation between parameters.

## Results

### Nano-emulsion specifications

**Fig. 1:**
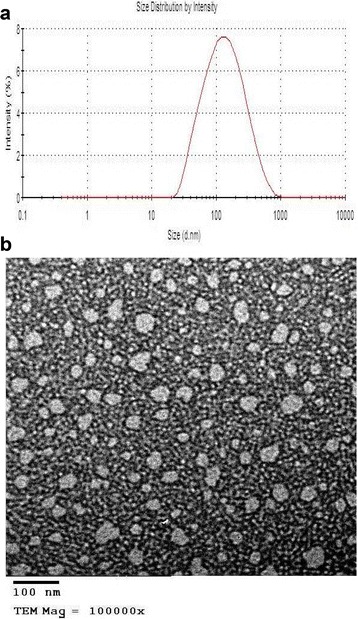
**a**. Particle size distribution of the prepared curcumin nano-emulsion measured using the zetasizer device. **b**. Transmission electron micrograph of the prepared curcumin nano-emulsion

The curcumin nano-emulsion was successfully prepared using the spontaneous emulsification method stated above. The prepared nano-emulsion showed a particle size of 141 ± 5.3 nm, a surface charge of −5.15 ± 0.98, and a polydispersity index of 0.369 ± 0.01, indicating its uniform particle size distribution. (Fig. 1a). The nanometer size was further confirmed by the transmission electron microscopy. (Fig. 1b).

### Effects of curcumin and curcumin nano-emulsion on body weight, testis weight, relative testis weight and sperm cell concentration

As shown in present data PDD resulted in a significant decrease in the rate of normal body weight increase, testis weight, relative testis weight as well as sperm cell concentration, % of normal sperm cell, mass motility and progressive motility%. Furthermore, the % of abnormal sperm cell was increased. On the other hand curcumin and curcumin nano-emulsion at high dose showed significant increase of final body weight, testis weight, relative testis weight in addition to sperm cell concentration, % of normal sperm cell, mass motility and progressive motility%. Besides, the % of abnormal sperm cell was reduced. Their results were comparable to each other. (Table 1).

**Table 1:** Effects of curcumin and curcumin nano-emulsion on body weight, testis weight, relative testis weight, Sperm cell concentration (×10^6^), % of normal and abnormal sperm cells, mass motility and progressive motility % of male rats under protein deficient diet model

Parameters	Groups				
	Normal	PDD	C	NCL	NCH
Initial body weight (gm)	61.1 ± 1.86	64 ± 1.83	62.6 ± 1.77	62.2 ± 1.78	60.3 ± 1.82
Final body weight (gm)	322 ± 10.51^b^	164 ± 5.31^a^	255 ± 7.92^ab^	177 ± 6.52^a^	209 ± 6.59^a, b^
Testis weight (gm)	1.68 ± 0.05^b^	0.4 ± 0.01^a^	1.101 ± 0.03^a, b^	0.53 ± 0.02^a, b^	0.802 ± 0.03^a, b^
Relative testis weight %	0.46 ± 0.01^b^	0.24 ± 0.01^a^	0.43 ± 0.01^b^	0.29 ± 0.01^a, b^	0.38 ± 0.01^a, b^
Sperm cell concentration × 10^6^ /gm cauda	144.0 ± 4.38^b^	44.30 ± 1.29^a^	114.1 ± 3.42^a, b^	58.7 ± 1.78^a, b^	98.7 ± 2.86^a, b^
% of normal sperm cell	69.82 ± 1.29^b^	29.70 ± 2.3^a^	52.47 ± 1.75^a, b^	40.84 ± 0.997^a, b^	52.18 ± 1.65^a, b^
% of abnormal sperm cell	30.17 ± 1.29^b^	70.29 ± 2.3a	47.52 ± 1.75^a, b^	59.15 ± 0.997^a, b^	47.82 ± 1.65^a, b^
Mass motility (Score)	3.73 ± 0.27^b^	1.53 ± 0.12^a^	2.77 ± 0.23^a, b^	1.84 ± 0.24^a^	2.67 ± 0.26^a, b^
Progressive motility%	58.34 ± 1.6^b^	23.01 ± 0.84^a^	39.73 ± 0.85^a, b^	32.19 ± 0.59^a, b^	39.71 ± 1.45^a, b^

^a^Significantly different from normal control, ^b^significantly different from PDD group

### Effect of curcumin and curcumin nano-emulsion on essential and non-essential amino acids levels

PDD significantly reduced all essential and non-essential amino acids levels as compared to normal control. Curcumin significantly elevated all essential amino acids levels including **L-arginine** as compared to PDD. Moreover; curcumin **normalized** most non-essential amino acids levels including taurine. Nano curcumin at the lower dose level significantly elevated most essential amino acids including **L-arginine** as compared to PDD control, the high dose significantly elevated **all essential amino acids** levels as compared to PDD control. Furthermore; nano curcumin at the lower dose level significantly elevated all non-essential amino acids levels including taurine as compared to PDD control and at the high dose **normalized** their levels. Comparing the results of curcumin and nano-curcumin at the high dose; it was easily demonstrated that nano-curcumin at the high dose showed superior results especially regarding both **L-arginine and taurine** (Table 2).

**Table 2:** Effects of curcumin and curcumin nano-emulsion on seminal plasma essential and nonessential amino acids of male rats under protein deficient diet model

Parameters (seminal plasma)		Groups				
		Normal	PDD	C	NCL	NCH
Essential amino acids nmol/ml	Arg	76.47 ± 0.67^b^	37.59 ± 0.85^a^	56.62 ± 1.104^a, b^	46.73 ± 0.42^a, b^	65.19 ± 1.36^a, b^
	His	34.27 ± 0.43^b^	17.93 ± 0.35^a^	25.45 ± 0.61^a, b^	21.09 ± 0.15^a, b^	27.11 ± 0.76^a, b^
	Isoleu	10.98 ± 0.16^b^	4.67 ± 0.012^a^	7.67 ± 0.12^a, b^	5.96 ± 0.07^a, b^	8.61 ± 0.11^a, b^
	Leu	17.13 ± 0.20^b^	8.12 ± 0.16^a^	12.71 ± 0.07^a, b^	9.61 ± 0.11^a, b^	13.28 ± 0.28^a,^ b
	Lys	54.46 ± 0.24^b^	27.03 ± 1.03^a^	38.19 ± 0.997^a, b^	34.39 ± 0.98^a, b^	44.24 ± 1.16^a, b^
	Met	23.15 ± 0.38^b^	11.90 ± 0.35^a^	17.02 ± 0.23^b^	12.45 ± 0.66^a^	17.14 ± 0.33^a, b^
	Val	9.94 ± 0.09^b^	4.413 ± 0.13^a^	6.83 ± 0.2^a, b^	5.50 ± 0.12^a, b^	7.49 ± 0.13^a, b^
Nonessential amino acids nmol/ml	Ala	238.8 ± 1.59^b^	157.7 ± 5.77^a^	234.5 ± 0.73^b^	204.5 ± 2.51^a, b^	287.8 ± 7.01^a, b^
	Asp	863.7 ± 9.98^b^	479.4 ± 7.55^a^	935.9 ± 9.91^a, b^	666.7 ± 22.03^a, b^	939.6 ± 4.22^a, b^
	Glu	295.6 ± 4.16^b^	161.4 ± 2.84^a^	296.2 ± 1.91^b^	237.8 ± 1.27^a, b^	349.6 ± 1.12^a, b^
	Gly	553.6 ± 8.32^b^	316.2 ± 7.87^a^	557.0 ± 14.47^b^	459.5 ± 12.23^a, b^	588.4 ± 8.69^b^
	Ser	786.5 ± 16.13^b^	416.3 ± 3.12^a^	657.2 ± 5.76^a, b^	591.8 ± 4.43^a, b^	788.1 ± 14.90^b^
	Tau	6.87 ± 0.11^b^	4.73 ± 0.03^a^	6.64 ± 0.01^b^	5.77 ± 0.13^a, b^	7.26 ± 0.20^b^
	Tyr	39.81 ± 0.82^b^	22.44 ± 0.08^a^	37.13 ± 0.35^b^	29.44 ± 0.34^a, b^	42.18 ± 1.16^b^

^a^Significantly different from normal control, ^b^significantly different from PDD group

### Effect of curcumin and curcumin nano-emulsion on serum testosterone level

**Fig. 2:**
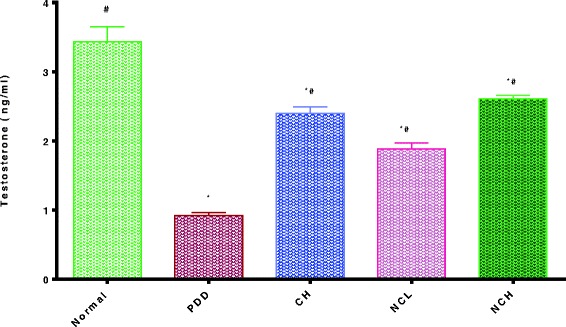
Effects of curcumin and curcumin nano-emulsion on serum free testosterone level of male rats under protein deficient diet model. *Significantly different from normal control, # significantly different from PDD group

PDD resulted in significant reduction in serum testosterone level (0.92 ± 0.04 vs. 3.43 ± 0.22 ng/ml) as compared to the normal control. Ingestion of the conventional dose of curcumin as well as nano-curcumin at both dose levels resulted in a significant elevation of the testosterone level (2.39 ± 0.1, 1.89 ± 0.08 and 2.61 ± 0.06 vs. 0.92 ± 0.04 ng/ml) respectively as compared to the PDD control; where the nano-curcumin at the higher dose level resulted in superior results over the conventional curcumin formula. (Fig. 2).

### Effect of curcumin and curcumin nano-emulsion on testicular tissue oxidative and nitrosative stresses parameters

PDD resulted in significant elevation of the MDA and NOx levels and GSSG/GSH ratio as well as significant reduction in the GSH level, CAT and SOD activities as compared to the normal control indicating extensive oxidative and nitrosative stresses. Ingestion of the conventional dose of curcumin (50 mg/kg) resulted in a significant decrease in the MDA and NOx levels and GSSG/GSH ratio in addition to a significant increase in the GSH content, CAT and SOD activities as compared to the PDD control. Administration of nano-curcumin at both dose levels also resulted in a pronounced anti-oxidant effect where the results of the higher dose were in most; superior over the conventional curcumin formula (Table 3).

**Table 3:** Effects of curcumin and curcumin nano-emulsion on testes oxidative and nitrosative stresses markers (MDA, NOx, GSH, GSSG, CAT and SOD) of male rats under protein deficient diet model

Parameters	Groups				
	Normal	PDD	C	NCL	NCH
GSH (μmol/g)	27.38 ± 0.43^b^	10.76 ± 0.60^a^	22.34 ± 0.68^a, b^	17.55 ± 0.57^a, b^	23.27 ± 0.68^a, b^
GSSG/GSH	0.0296 ± 0.0002^b^	0.208 ± 0.0143^a^	0.0486 ± 0.0019^b^	0.0713 ± 0.0031^a, b^	0.0436 ± 0.0018^b^
MDA (nmol/g)	16.91 ± 0.55^b^	49.29 ± 0.24^a^	24.44 ± 0.73^a, b^	27.84 ± 0.14^a, b^	20.46 ± 0.49^a, b^
NOx (μmol/g)	2.10 ± 0.05^b^	5.85 ± 0.26^a^	3.04 ± 0.09^a, b^	3.11 ± 0.11^a, b^	2.48 ± 0.09^b^
CAT (U/mg protein)	29.36 ± 0.35^b^	9.58 ± 0.25^a^	14.94 ± 0.14^a, b^	19.53 ± 0.4^a, b^	19.82 ± 0.42^a, b^
SOD (U/mg protein)	1858 ± 32.47^b^	406.6 ± 5.808^a^	942.8 ± 28.67^a, b^	1129 ± 5.461^a, b^	1144 ± 9.835^a, b^

^a^Significantly different from normal control, ^b^significantly different from PDD group

### Effect of curcumin and curcumin nano-emulsion on testicular tissue 8- OHDG level

**Fig. 3:**
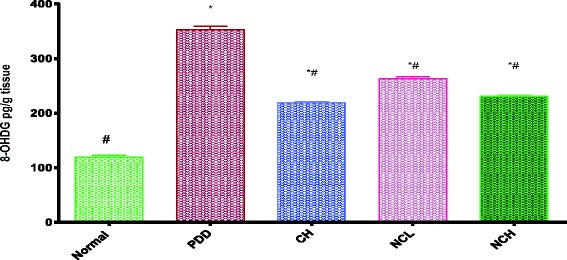
Effects of curcumin and curcumin nano-emulsion on testes 8OHDG concentration of male rats under protein deficient diet model. *Significantly different from normal control, # significantly different from PDD group

PDD resulted in significant elevation in testicular tissue 8-hydroxy-2-deoxyguanosine (8-OHDG) level (352.6 ± 6.30 vs. 119.3 ± 3.64 pg/g) as compared to the normal control. Ingestion of the conventional dose of curcumin as well as nano-curcumin at both dose levels resulted in a significant reduction in the testicular tissue 8-OHDG level (218.9 ± 2.42, 263.1 ± 3.97 and 231.4 ± 1.21 vs. 352.6 ± 6.30 pg/g) respectively as compared to the PDD control indicating DNA preservation (Fig. 3).

### Effect of curcumin and curcumin nano-emulsion on testicular tissue cell energy performance

PDD resulted in significant reduction in cell energy represented by decreased adenylate energy charge (AEC) and increased AMP/ATP ratio as compared to normal control. Ingesting the conventional dose of curcumin as well as nano-curcumin at both dose levels reversed that disruption in cell energy; normalizing the AMP/ATP ratio and the AEC, where the higher dose on nano-curcumin showed superior results over the conventional curcumin formula (Table 4).

**Table 4:** Effects of curcumin and curcumin nano-emulsion on testes cell energy (ATP, ADP, AMP, AMP/ATP and ACE) of male rats under protein deficient diet model

Parameters	Groups				
	Normal	PDD	C	NCL	NCH
ATP (μg/g)	85.68 ± 1.91^b^	19.35 ± 0.38^a^	48.47 ± 0.45^a, b^	57.56 ± 0.32^a,m b^	64.84 ± 0.66^a, b^
ADP (μg/g)	36.42 ± 0.66^b^	6.95 ± 0.23^a^	19.15 ± 0.50^a,b^	23.23 ± 0.50^a, b^	22.62 ± 0.21^a, b^
AMP (μg/g)	26.79 ± 0.48^b^	8.97 ± 0.24^a^	15.94 ± 0.08^a, b^	17.07 ± 0.52^a, b^	17.55 ± 0.30^a, b^
AMP/ATP	0.31 ± 0.002^b^	0.46 ± 0.014^a^	0.33 ± 0.007^b^	0.30 ± 0.006^b^	0.27 ± 0.002^a, b^
AEC	0.70 ± 0.002^b^	0.65 ± 0.012^a^	0.69 ± 0.006^b^	0.71 ± 0.003^b^	0.73 ± 0.006^a, b^

^a^Significantly different from normal control, ^b^significantly different from PDD group

### Correlation studies

**Fig. 4:**
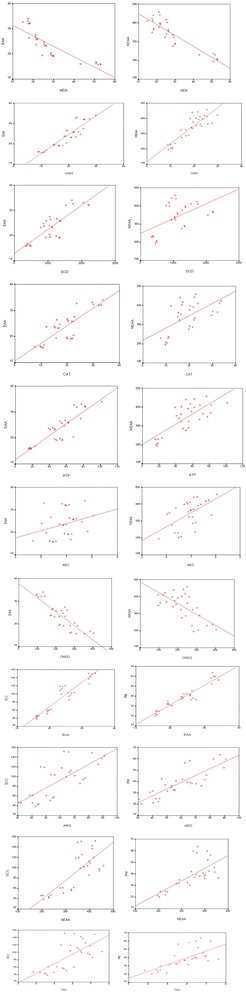
Correlation graphs

Our results revealed positive correlation between the increased essential & non-essential amino acids contents in the seminal plasma and the increased seminal plasma sperm cell concentration & progressive sperm motility also there was a positive correlation between the levels of L- arginine & taurine and the increased seminal plasma sperm cell concentration & progressive sperm motility. Moreover the incidence of testicular tissue oxidative stress, DNA damage and disrupted cell energy turned to be negatively correlated to the essential and non-essential amino acids contents in the seminal plasma (Table 5, Fig. 4).

**Table 5:** Pearson’s correlations studies

	MDA	GSH	SOD	CAT	ATP	AEC	8-OHDG	EAA	ARG	NEAA	TAU	SCC	PM
MDA	**NA**	−0.865^***^	−0.790^**^	−0.740^**^	−0.851^***^	−0.387	0.840^***^	−0.852^***^	−0.779^**^	−0.894^***^	−0.806^***^	−0.808^***^	−0.827^***^
GSH	−0.865^***^	**NA**	0.784^**^	0.825^***^	0.907^***^	0.566^*^	−0.710^**^	0.909^***^	0.673^**^	0.866^***^	0.651^**^	0.871^***^	0.853^***^
SOD	−0.790^**^	0.784^**^	**NA**	0.887^***^	0.836^***^	0.212	−0.795^**^	0.846^***^	0.685^**^	0.535^*^	0.623^**^	0.745^**^	0.884^***^
CAT	−0.740^**^	0.825^***^	0.887^***^	**NA**	0.941^***^	0.402^*^	−0.690^**^	0.839^***^	0.609^**^	0.591^*^	0.494^*^	0.707^***^	0.814^***^
ATP	−0.851^***^	0.907^***^	0.836^***^	0.941^***^	**NA**	0.536^*^	−0.695^**^	0.874^***^	0.649^**^	0.762^**^	0.577^*^	0.744^***^	0.821^***^
AEC	−0.387	0.566^*^	0.212	0.402^*^	0.536^*^	**NA**	0.016	0.298	0.034	0.570^*^	0.030	0.202	0.217^*^
8-OHDG	0.840^***^	−0.710^**^	−0.795^**^	−0.690^**^	−0.695^**^	−0.016	**NA**	−0.867^***^	−0.776^**^	−0.663^**^	−0.719^**^	−0.885^***^	−0.871^***^
EAA	−0.852^***^	0.909^***^	0.846^***^	0.839^***^	0.874^***^	0.298	−0.867^***^	**NA**	0.825^***^	0.793^**^	0.715^**^	0.939^***^	0.953^***^
ARG	−0.779^**^	0.673^**^	0.685^**^	0.609^**^	0.649^**^	0.034	−0.776^**^	0.825^***^	**NA**	0.698^**^	0.844^***^	0.730^**^	0.829^***^
NEAA	−0.894^***^	0.866^***^	0.535^*^	0.591^*^	0.762^**^	0.570^*^	−0.663^**^	0.793^**^	0.698^**^	**NA**	0.718^**^	0.772^**^	0.701^**^
TAU	−0.806^***^	0.651^**^	0.623^**^	0.494^*^	0.577^*^	0.030	−0.719^**^	0.715^**^	0.844^***^	0.718^**^	**NA**	0.677^**^	0.672^**^
SCC	−0.808^***^	0.871^***^	0.745^**^	0.707^**^	0.744^**^	0.202	−0.885^***^	0.939^***^	0.730^**^	0.772^**^	0.677^**^	**NA**	0.910^***^
PM	−0.827^***^	0.853^***^	0.884^***^	0.814^***^	0.821^***^	0.217	−0.871^***^	0.953^***^	0.829^***^	0.701^**^	0.672^**^	0.910^***^	**NA**

Negative correlation **(−)**, significant correlation at (*p* < 0.05) *, (*p* < 0.01) **, (*p* < 0.001) ***, non-applicable correlation **(NA)**

**Fig. 5:**
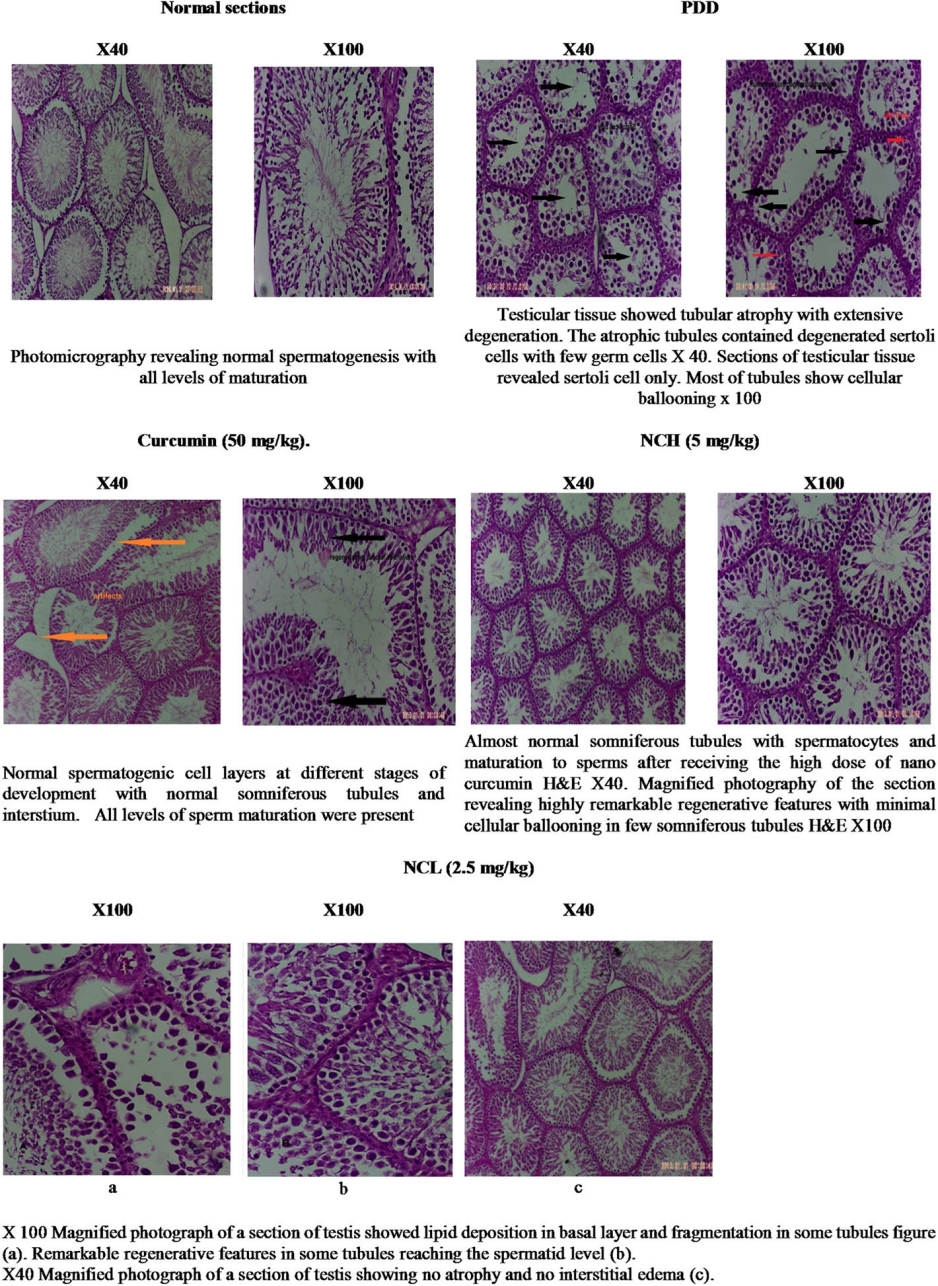
Histopathological examination

### Histopathological examination

H& E staining for the group ingesting protein deficient diet (PDD diet) for 75 days revealed tubular atrophy with extensive degeneration in testicular tissue. The atrophic irregular seminiferous tubules X40 were characterized by a depletion of germ cells, exhibiting sertoli cell-only type and a few spermatogonia. Reduced seminiferous epithelial layers were found in numerous tubules, and irregular and diminished tubules containing a few germ cells were also seen X100. Ingesting curcumin at the conventional dose level resulted in normal spermatogenic cell layers at different stages of development with normal seminiferous tubules and interstium, all levels of sperm maturation were present. Examining sections from the group ingesting nano-curcumin at the low dose level revealed remarkable regenerative features in all the tubules reaching the spermatid level but lipid deposition in the basal layer could be observed at some tubules figure (a) and fragmentation in some tubules (b) H&E X100. Normal gaps between seminiferous tubules is preserved with no atrophy or interstitial edema. On the other hand; ingesting nano-curcumin at the high dose level resulted in sections with almost reduced seminiferous epithelial layers in numerous tubules with maturation to sperms H&E X100. Magnified photography of the section revealing highly remarkable regenerative features with cellular ballooning in few seminiferous tubules with diminished normal intratubular gaps relatively higher than group of low dose level. H&E X100. (Fig. 5). Furthermore; morphometric measures revealed that PDD significantly reduced the mean seminiferous tubules circumferences and total sperm cells maturation count as compared to the normal control. On the other hand; ingesting curcumin and curcumin nano-emulsion at the two dose levels significantly increased both the mean tubules circumferences and total sperm cells maturation count indicating enhancement in the overall spermatogenesis process. According to Modified Johnsen spermatogenesis scoring; taking in consideration that Johnsen scoring is covering only the spermatogenic leveling in testicular biopsy away from other pathological features that could be detected; it was clearly demonstrated that the normal control group showed a score of 10 while the PDD control scored 2. Ingesting curcumin in its conventional form resulted in retrieving the score of 10. On the other hand; administration of nano-curcumin in the lower dose level showed mixed scores of 8 & 9 according to captures from different sections. Moreover; ingesting curcumin nano-emulsion at the higher dose level displayed score of 8 & 9 at some fields and 10 at other. This result further supports our findings and prove the effectiveness of curcumin nano-emulsion (Table 6).

**Table 6:** Effects of curcumin and curcumin nano-emulsion on total sperm cells maturation count and seminiferous tubules circumferences of male rats under protein deficient diet model

Groups	Total sperm cells maturation (count)	seminiferous tubules circumferences (μm)	Modified Johnsen spermatogenesis scoring
Normal	290.5 ± 5.14^b^	198.6 ± 4.94^b^	10.00 ± 0.0^b^
PDD	8.875 ± 0.72^a^	90.87 ± 1.44^a^	2.00 ± 0.0^a^
C	245.0 ± 5.09^a, b^	235.2 ± 7.68^a, b^	10.00 ± 0.0^b^
NCL	241.9 ± 3.52^a, b^	166.1 ± 4.10^a, b^	8.88 ± 0.3^a^
NCH	244.6 ± 5.26^a, b^	143.3 ± 5.36^a, b^	9.5 ± 0.19^b^

^a^Significantly different from normal control, ^b^significantly different from PDD group

## Discussion

Curcumin, a component of the spice turmeric (*Curcuma longa*), is widely used as a traditional herbal medicine in the treatment of various diseases. This compound can inhibit oxidative stress and could ameliorate tissue atrophy [37]. Previous investigators reported the beneficial effects of ingesting curcumin on testicular tissue; both in acute and chronic models of testicular tissue injury; which could be clearly confirmed on both histological level and biochemical parameters estimated; suggesting the possibility of using curcumin as a potential therapeutic in the treatment of stress-mediated testicular dysfunction. Treatment with curcumin markedly decreased apoptosis in rat testes, increased the mean seminiferous tubule diameter and mean testicular biopsy score values. A significant reduction in the testicular tissue oxidative stress and decrease in sperm abnormalities as well as increased total sperm count and elevation in testosterone level were demonstrated [6, 38–40].

In the present investigation protein deficient diet (PDD) based on ingesting pellets of corn grains was used to create a state of malnutrition in rats. This diet was used according to previous researches indicating that corn grains contained very low protein content as compared to the recommended protein contents in standard rat diet [15, 41, 42]. Former investigators reported that sperm maturation needs about 70 days in rats [16]; therefore, rats were given the diet for 75 days and the drug treatments were administered daily during the last 15 days of the diet regimen.

The relationship between reproduction and malnutrition has been studied in laboratory animals by employing diets deficient in or totally devoid of protein. It has been demonstrated that under-nutrition during the fetal and/or pre-pubertal period is accompanied by changes in testicular structure with a consequent decrease in daily sperm production [1–3]. Besides; supplementation of diet with amino acids improved sperm quality, and subsequently increased fertilization capacity [43]. Furthermore; adequate dietary intake of protein in general is considered essential for maintenance of adequate tissue adenylate energy charge (AEC) and increased ATP production in all body organs [44].

With respect to redox signaling, it has recently been demonstrated that reactive oxygen and nitrogen species (ROS and RNS) and the resultant oxidative and nitrosative stresses are implicated in male infertility. When the oxidative and nitrosative stresses exceed the antioxidant capacity of any body tissue an extensive amount of malondialdehyde (MDA) is generated, intense cellular damage and consequential accelerated apoptosis follows. The overall status affects tissue DNA and membrane phospholipids integrity; resulting in severe tissue injury and/or cell death [45–47]. 8-hydroxylated guanine species such as 8-oxoguanine and 8-hydroxy-2-deoxyguanosine (8-OHDG) are repair products of oxidized guanine lesions. 8-OHDG content is considered a sensitive biomarker of the oxidative DNA damage and repair [48]. Besides the superoxide anion is the main undesired by-product of mitochondrial oxidative phosphorylation. Superoxide dismutase (SOD) converts superoxide anion to hydrogen peroxide, which can be then converted by catalase (CAT) to harmless H_2_O [49].

During malnutrition and protein deficiency, there is excess production ROS and RNS. Furthermore; it was demonstrated that diets deficient in methionine are usually accompanied by progressive increase in MDA and NOx levels as well as pronounced reduction in GSH level along with marked decrease in CAT and SOD activities. Taking into consideration that necessity of GSH, CAT and SOD for body tissues as defense strategy against free radical damage; preserving cellular content of those antioxidant moieties could be critical for maintaining optimum health and wellbeing [46, 50–52].

Semen consists of spermatozoa suspended in a fluid medium called seminal plasma; which is a complex fluid portion and mediates the chemical function of the ejaculate. Biochemical components of seminal plasma are synthesized and secreted by the rete testis, epididymis, and accessory sex glands of the male reproductive tract. The conventional role for seminal plasma is acting as a survival medium that facilitates transport of spermatozoa. Generally; seminal plasma plays important roles in increasing the overall sperm quality parameters, activation and augmentation of the motility of spermatozoa as well as buffering to provide the optimal osmotic and nutrient medium. Moreover; it has also been observed that the use of preserved semen for artificial insemination in livestock species, which often involves extensive dilution or removal of seminal plasma, results in lower fertility rates than with natural mating. These evidences suggest that seminal plasma components participate in key events related to sperm function, fertilization, and embryo development in the female reproductive tract. Therefore; the role of seminal plasma constituents in regulating sperm functions must be highlighted in reproductive investigations. Seminal plasma amino acids in particular; serve as a readily oxidizable substrate for energy-yielding reactions in semen to fuel the progressive and hyperactive motility of sperms; necessary for capacitation and fertilization. One of the most important essential amino acids involved in the energy production process would be L-Arginine which acts as a source of energy for normal sperm motility in the form of arginine phosphoric acid. In addition; taurine has been identified as one of the major free amino acids of the seminal fluid. Several physiological functions of taurine have been demonstrated, such as membrane stabilization, sperm motility factor, energy storage and acting as an anti-oxidant [53, 54].

That is why our research was based on examining the effect of PDD on the testicular tissue as well as the spermatogenesis process, sperm motility and seminal plasma essential and non-essential amino acids content with special focus on the levels of both L-arginine and taurine.

The results of the current study clearly showed that PPD resulted in severe reduction in the rate of total body weight increase as well as marked decrease in testes weight, relative testes weight, total sperm count, mass and progressive motilities. On biochemical level; testicular tissue adenylate energy charge (AEC) was lowered, pronounced increase in 8-OHDG level and marked oxidative and nitrosative stresses in the testicular tissue were demonstrated. Serum testosterone level as well as several seminal plasma essential and non-essential amino acids levels were reduced and generally troubled spermatogenesis was definite. Histopathological examination revealed the existence of testicular tissue atrophy and degeneration as well as alterations in the whole spermatogenesis process.

Furthermore, our results also revealed the existence of a negative correlation between testicular tissue elevated level of 8-OHDG, increased oxidative stress as well as alteration in cell energy and the levels of both essential and non-essential amino acids in the seminal plasma as well as sperm cell concentration and progressive sperm motility. It was also demonstrated that the levels of essential and non-essential amino acids and in particular L-arginine and taurine were positively correlated to the sperm cell concentration and progressive sperm motility.

Our results revealed that ingesting curcumin and nano-curcumin protected against the adverse effects of PDD. Curcumin and nano-curcumin resulted in elevated rate in total body weight increase as well as marked increase in testes weight, relative testes weight, testicular tissue cell energy in addition to pronounced decrease in testicular tissue 8-OHDG level as well as decreased oxidative and nitrosative stresses. Furthermore; there was a pronounced elevation in serum testosterone level, as well as seminal plasma essential and non-essential amino acids content specially L-arginine and taurine. Regarding spermatogenesis process there was a marked increase in the total sperm cell concentration, mass and progressive sperm motility and sperm viability and normality. Histopathological examination demonstrated normal spermatogenesis with remarkable regenerative features in seminiferous tubules with no atrophy or interstitial edema. The steroidogenic effect of curcumin may be due to the indirect increase of testosterone level through blocking the metabolism of testosterone. The mechanism by which curcumin has been considered to be a testosterone-increasing agent is by acting as an aromatase inhibitor; an enzyme that converts testosterone to estrogen [55].

The superiority of curcumin nano-emulsion compared to mere curcumin; as manifested by high therapeutic efficacy at a much lower dose could be attributed to the reported internalization of nano-emulsion droplets into the enterocytes by clathrin-mediated endocytosis pathway, which would allow the curcumin loaded nano-emulsion to be transported into the systemic circulation by the portal vein and lymphatic pathway [56]. Furthermore, the co-ingestion of a lipid source; in our case the oil of the nano-emulsion, was reported to enhance the bioaccessibility of lipophilic compounds by increasing their solubility in gastrointestinal tract (GIT) fluids, as the lipids stimulate the production of digesting enzymes as well as bile salts. Moreover, the nano size of the emulsion is postulated to have induced higher lipid hydrolysis rate leading to higher bioaccessibility of curcumin, thus consequently leading to bioavailability enhancement [57].

## Conclusion

As can be inferred from the previous results that both curcumin and curcumin in nano-emulsion form managed to exhibit favorable outcomes regarding the reproductive profile in rats indicated by testicular tissue cell energy preservation, assistance in DNA repair as well as improvement of seminal plasma quality as confirmed by the amino acids profile and improvement in the overall spermatogenesis process. Curcumin in nano-emulsion proved to be more superior in several aspects, owing to nanometer size, solubilizing potential for curcumin, and lipophilicity leading to enhanced bioavailability. Futuristic studies should focus on more possible beneficial effects from using curcumin in nano-emulsion on other male fertility parameters and the advantageous consequences from using such nano-emulsion form of curcumin on sperm-egg interaction and fertilization.

## Abbreviations

8-OHDG: 8-hydroxy-2-deoxyguanosine
ADP: Adenosine diphosphate
AEC: Adenylate energy charge
Ala: Alanine
AMP: Adenosine monophosphate
Arg: L-arginine
Asp: Aspartate
ATP: Adenosine triphosphate
C: Curcumin group
CAT: Catalase
Glu: Glutamate
Gly: Glycine
GSH: Reduced glutathione
GSSG: Oxidized glutathione
H&E: Hematoxylin and Eosin
His: Histidine
Isoleu: Isoleucine
Leu: Leucine
Lys: Lysine
MDA: Malondialdehyde
Met: Methionine
NCH: Curcumin nano-form high dose group
NCL: Curcumin nano-form low dose group
NOx: Nitric oxide nitrate and nitrite forms
PDD: Protein deficient diet
RNS: Reactive nitrogen species
ROS: Reactive oxygen
Ser: Serine
SOD: Superoxide dismutase
Tau: Taurine
Tyr: Tyrosine
Val: Valine
